# Repetitive Transcranial Magnetic Stimulation Combined With Median Nerve Electrical Stimulation for Severe Traumatic Brain Injury

**DOI:** 10.1002/brb3.71661

**Published:** 2026-09-28

**Authors:** Nana Wang, Manrong Li, Guozhen Zhang

**Affiliations:** ^1^ Rehabilitation Department Shanghai Xinqidian Rehabilitation Hospital Shanghai China; ^2^ Department of Rehabilitation Shanghai East Hospital Tongji University Shanghai China; ^3^ Rehabilitation Department Pudong Hospital Shanghai China

**Keywords:** disorders of consciousness, median nerve stimulation, repetitive transcranial magnetic stimulation, severe traumatic brain injury, transcranial Doppler

## Abstract

**Background:**

Severe traumatic brain injury (sTBI) often leads to prolonged coma and poor neurological outcomes. Noninvasive neuromodulation techniques such as repetitive transcranial magnetic stimulation (rTMS) and median nerve electrical stimulation (MNS) have shown potential in promoting arousal. However, the efficacy of combining these two approaches remains unclear.

**Methods:**

In this randomized controlled trial, 86 patients with sTBI‐induced coma were assigned to rTMS (*n* = 29), MNS (*n* = 29), or combination therapy (*n* = 28) for 4 weeks. Outcomes included awakening efficacy, EEG grading, cerebral blood‐flow parameters (Vm, Vd, Vs, pulsatility index [PI]), GCS, and MoCA. Safety events were recorded.

**Results:**

At week 4, the combination group demonstrated significantly higher overall awakening efficacy (92.86%, 26/28) compared with the rTMS (65.52%, 19/29) and MNS (62.07%, 18/29) groups (*p* < 0.05; number needed to treat [NNT] ≈ 4 for combination vs. MNS). GCS scores improved significantly within all groups (all *p* < 0.05), with the greatest mean change observed in the combination group (baseline 5.19 ± 0.62 → week 4: 11.23 ± 1.12; Δ+6.04), followed by MNS (Δ+4.50) and rTMS (Δ+4.19); between‐group differences favored combination therapy (*p* < 0.05). EEG grading shifted toward lower‐severity categories in all groups, with the combination group achieving the highest proportion of Grade I–II normalization at week 4 (71.4%, 20/28) compared with rTMS (62.1%, 18/29) and MNS (37.9%, 11/29). Transcranial Doppler–derived cerebral blood‐flow velocities (Vm, Vs, Vd) increased and the pulsatility index decreased across all groups, with the combination group demonstrating the most pronounced hemodynamic improvement relative to either monotherapy (all *p* < 0.05). In an exploratory analysis restricted to patients who regained sufficient responsiveness to undergo cognitive testing (combination: 26/28 [92.9%]; rTMS: 19/29 [65.5%]; MNS: 18/29 [62.1%]), MoCA scores at week 4 indicated mild‐to‐moderate cognitive impairment across all groups (combination: 21.35 ± 2.87; rTMS: 18.74 ± 3.26; MNS: 18.22 ± 3.41), with numerically highest scores in the combination group; however, given the differential testability rates—which corresponded directly to awakening status—these findings are subject to inherent selection bias and should be interpreted as hypothesis‐generating only. No seizures were observed throughout the study period. Adverse events were infrequent and did not differ significantly among groups (combination: 10.7%; rTMS: 20.7%; MNS: 27.6%; all pairwise *p* > 0.05), with rates numerically lowest in the combination group.

**Conclusions:**

In patients with severe traumatic brain injury and persistent coma, rTMS combined with right MNS significantly improves awakening efficacy, neurophysiological indicators (EEG grading), and cerebral hemodynamics compared with either modality alone. GCS improvement was greatest with combination therapy. Exploratory cognitive findings (MoCA in testable subsets) suggest potential additional benefits, though these require confirmation in future trials employing outcome instruments applicable to all randomized patients. Adverse event rates were low across all groups, with no seizures observed; the study was not powered for formal safety comparisons. These findings support the dual‐pathway neuromodulation paradigm as a promising strategy for consciousness recovery in sTBI and warrant confirmation in multicenter, sham‐controlled trials with extended follow‐up.

## Graphical abstract

Combining top‐down rTMS over the left DLPFC with bottom‐up right median nerve stimulation markedly outperformed either modality alone, awakening 93% of patients with severe TBI by week 4 and improving EEG, hemodynamics, and GCS—supporting dual‐pathway neuromodulation as a safe, high‐efficacy strategy for coma recovery.

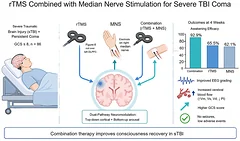

**Abbreviations**
EEG: electroencephalogram
GCS: Glasgow Coma Scale
MNS: median nerve stimulation
MoCA: Montreal Cognitive Assessment
PI: pulsatility index
RMT: resting motor threshold
rTMS: repetitive transcranial magnetic stimulation
sTBI: severe traumatic brain injury
TCD: transcranial Doppler

## 1 Introduction

Severe traumatic brain injury (sTBI) represents a major global public health challenge, contributing to substantial long‐term disability, mortality, and socioeconomic burden worldwide (Iaccarino et al. 2018; Yan et al. 2022). Caused primarily by direct external mechanical forces that produce serious structural and functional brain damage (Wang et al. 2023), sTBI remains one of the leading causes of death and long‐term disability among young adults globally, with a disproportionate burden increasingly recognized in low‐ and middle‐income countries (Zhao et al. 2016). Dewan et al. (2019) conducted a comprehensive global analysis estimating that 69 million individuals suffer TBI annually worldwide. Survivors of sTBI frequently experience prolonged disorders of consciousness (DoC)—a clinical spectrum encompassing coma, vegetative state/unresponsive wakefulness syndrome (VS/UWS), and minimally conscious state (MCS)—characterized by severely impaired awareness, absent or near‐absent purposeful behavior, and profoundly limited neurological recovery, ultimately leading to profound loss of independence and quality of life (Li et al. 2018; Schnakers and Monti 2017). The personal, familial, and societal costs of these conditions are substantial: patients may remain in states of severely impaired awareness for months to years, placing extraordinary demands on caregiving systems and healthcare resources worldwide. Despite meaningful advances in neurocritical care and neurorehabilitation, conventional pharmacologic and supportive strategies frequently yield suboptimal outcomes in terms of consciousness recovery, underscoring the urgent need for novel, evidence‐based interventions to promote recovery of consciousness in this population.

The clinical urgency of this challenge cannot be overstated. To date, no single pharmacologic or neuromodulatory intervention has achieved sufficient evidence to be considered a standard of care for DoC following sTBI. Noninvasive brain stimulation (NIBS) has emerged as a promising therapeutic approach to modulate large‐scale neural networks underlying arousal and awareness (Cossu 2014). Among pharmacologic interventions, amantadine—an N‐methyl‐d‐aspartate receptor antagonist and dopaminergic agent—has been shown to accelerate functional recovery on standardized disability scales in selected post‐traumatic DoC patients, whereas zolpidem may transiently enhance arousal in a small subset through a paradoxical GABAergic mechanism; however, the effects of both agents are variable, reliable predictive biomarkers are lacking, and standardized treatment protocols remain elusive (Schnakers and Monti 2017; Whyte and Myers 2009) Within the spectrum of NIBS modalities, transcranial direct current stimulation (tDCS) currently has the greatest number of formally designed randomized trials in DoC, yet the overall quality and consistency of evidence remain limited by small sample sizes, methodological heterogeneity, and inconsistent outcome operationalization (Li et al. 2015; Neville et al. 2019; Thibaut et al. 2014). These limitations collectively highlight the need for rigorously designed trials of alternative or complementary neuromodulatory strategies.

Repetitive transcranial magnetic stimulation (rTMS) offers a biologically appealing option for patients with DoC because it can noninvasively modulate cortical excitability and promote functional connectivity within consciousness‐related networks, supporting synaptic plasticity and large‐scale network reorganization (Hallett 2007; Huang et al. 2005). Preclinical studies suggest potential neuroprotective and pro‐recovery effects, but clinical evidence in humans remains constrained by small, heterogeneous samples and varying injury characteristics (Nardone et al. 2020). Early clinical investigations indicate that rTMS is generally well tolerated and may improve neurobehavioral responsiveness. For instance, case‐level studies have documented improvements in consciousness and electrophysiological markers, such as brainstem auditory evoked potentials, without notable adverse events during multi‐week stimulation protocols (Louise‐Bender Pape et al. 2009). Moreover, a randomized controlled trial demonstrated that left dorsolateral prefrontal cortex (DLPFC) stimulation enhanced consciousness scores compared with sham in some—but not all—patients (Louise‐Bender Pape et al. 2009). A recent systematic review and meta‐analysis further confirmed that high‐frequency rTMS protocols over the left DLPFC are associated with significant improvements in consciousness‐related outcomes, although effect sizes remain heterogeneous across studies and patient populations (Yang et al. 2023). Collectively, these findings support further investigation while highlighting the need to determine optimal stimulation parameters, cortical targets, and candidate populations.

Median nerve stimulation (MNS), a peripheral neuromodulation technique, is hypothesized to activate ascending reticular activating pathways and thalamo‐cortical circuits, thereby facilitating cortical arousal through a bottom‐up mechanism (Zhong et al. 2015). Right‐sided MNS has been specifically proposed to preferentially engage vigilance‐related networks of the non‐dominant hemisphere, providing a neuroanatomical basis for its selective use in arousal promotion (Zhong et al. 2015). Conceptually, integrating central (rTMS) and peripheral (MNS) stimulation may produce synergistic network effects by coupling top‐down cortical facilitation with enhanced subcortical drive, thereby accelerating the network reorganization required for emergence from coma (Du et al. 2022). However, comparative, controlled evidence evaluating this combination therapy in sTBI‐related DoC remains scarce, and no adequately powered three‐arm randomized trial has directly isolated the added value of combination therapy over either monotherapy alone.

The present study addresses this specific and clinically important evidence gap. Noninvasive neuromodulation strategies are particularly appealing in the context of sTBI‐related DoC because they are relatively low‐cost, do not require surgical intervention, and can be initiated during the acute‐to‐subacute rehabilitation phase when activity‐dependent neural plasticity is presumed to be highest (Huang et al. 2005; Li et al. 2018; Thibaut et al. 2014). If combining central and peripheral stimulation can reliably accelerate consciousness recovery, this would provide proof‐of‐concept for multimodal neuromodulation as a therapeutic paradigm with immediate implications for clinical practice, rehabilitation unit protocols, and future trial design. Clinicians and researchers with interests in neurorehabilitation, brain stimulation, disorders of consciousness, and neurocritical care will find these findings directly relevant to both their practice and investigative priorities. Accordingly, we conducted a randomized controlled trial to compare the efficacy and safety of rTMS, MNS, and their combination in patients with sTBI and persistent coma. The primary outcome was awakening efficacy at 4 weeks. Secondary outcomes included neurophysiological and functional assessments—electroencephalographic (EEG) grading, transcranial Doppler–derived cerebral blood flow parameters, Glasgow Coma Scale (GCS), and, in patients who regained responsiveness, the Montreal Cognitive Assessment (MoCA)—as well as adverse events. This study aims to determine whether integrating central and peripheral noninvasive neuromodulation yields superior clinical benefits compared with either modality alone during early neurorehabilitation following severe traumatic brain injury.

## 2 Methods

### 2.1 Study Design and Participants

This prospective, randomized controlled trial was conducted at Shanghai Xinqidian Rehabilitation Hospital between January 2023 and December 2024. Eighty‐six patients with severe traumatic brain injury (sTBI) and persistent coma were enrolled and randomized to rTMS (*n* = 29), MNS (*n* = 29), or combination therapy (*n* = 28) using a computer‐generated sequence.

Sample size calculation: The required sample size was estimated a priori using awakening efficacy at week 4 as the primary endpoint. The assumed monotherapy awakening rate of approximately 60% was derived from a prospective internal pilot cohort of 18 sTBI patients treated at our center (October–December 2022; rTMS: 6/9 [66.7%]; MNS: 5/9 [55.6%]; mean 61.1%), consistent with the neurobehavioral improvements reported in prior rTMS (Yang et al. 2023) and MNS (Feller et al. 2021) systematic reviews. The assumed combination therapy awakening rate of approximately 85% was informed by the same pilot cohort (8/9 [88.9%]) and supported by the trial of Xiong et al. (2023), which demonstrated superior consciousness outcomes with combined rTMS and MNS. We acknowledge that these assumptions relied predominantly on small‐sample pilot data, which is a recognized limitation of the study design. Based on these assumptions, sample size was calculated for a three‐group *χ* ^2^ test (df = 2) with a two‐sided significance level of *α* = 0.05 and a target statistical power of 1 − *β* = 0.80, yielding a minimum of 26 patients per group (78 total). After applying a 10% inflation factor to account for potential dropout and protocol deviations, the final enrollment target was set at 86 patients. The unequal allocation of 29, 29, and 28 patients to the rTMS, MNS, and combination groups, respectively, resulted from the block randomization sequence and the fixed total sample of 86, which is not evenly divisible by three. Sample size calculations were performed using PASS 15.0 software (NCSS, LLC, Kaysville, UT, USA). For transparency, the estimated post hoc statistical power based on the observed primary outcome effect (RR = 1.50, RD = +30.8% for combination vs. MNS) was approximately 87%. However, post hoc power calculations are a direct function of the observed *p*‐value and do not provide independent confirmation of study adequacy; the a priori calculation remains the primary basis for evaluating whether the study was adequately powered.

Inclusion criteria: (1) sTBI diagnosed by clinical and radiological criteria; (2) GCS ≤ 8; (3) coma duration > 24 h; (4) written informed consent from legal representatives.
Exclusion criteria: (1) severe pre‐existing neurological or psychiatric disorders; (2) severe cardiac, hepatic, or renal dysfunction; (3) contraindications to electrical or magnetic stimulation (e.g., pacemaker, intracranial metallic implants).

Allocation concealment used opaque, sequentially numbered, sealed envelopes prepared by an independent statistician. EEG/TCD/GCS/MoCA assessments were performed by evaluators blinded to treatment assignment.

### 2.2 Interventions

rTMS: rTMS was administered using the CCY‐I Transcranial Magnetic Stimulator (Wuhan Yiruide Medical Equipment Co., Ltd., Wuhan, China) equipped with a 70‐mm figure‐8 coil. Prior to commencing each treatment week, the resting motor threshold (RMT) was individually determined for each patient as the minimum stimulator output intensity capable of eliciting a visible motor‐evoked potential of ≥50 µV in the contralateral first dorsal interosseous muscle in at least 5 of 10 consecutive pulses. Stimulation was then delivered at 80% of the individually determined RMT, at a frequency of 10 Hz, organized in 5‐s trains separated by 25‐s inter‐train intervals, yielding 2000 pulses per session over approximately 20 min. Treatment was administered 5 days per week for 4 consecutive weeks. The stimulation target was the left dorsolateral prefrontal cortex (DLPFC), localized using the F3 electrode position of the international 10–20 system.

MNS: MNS was delivered via surface electrodes placed over the right median nerve at the wrist. Stimulation parameters were set at 20 Hz with a pulse width of 300 µs. The stimulation current was individually titrated to the minimum intensity sufficient to elicit clearly visible thumb abduction, while remaining strictly below the threshold for nociceptive withdrawal responses, ensuring patient comfort and tolerability throughout treatment. Sessions lasted 20 min and were conducted 5 days per week for 4 weeks, identical in schedule to the rTMS protocol.

Combination: Patients assigned to the combination group received sequential central and peripheral neuromodulation: rTMS was administered first, followed within 10 min by right‐sided MNS. Both modalities were applied using the identical parameters and weekly schedule described above for each respective monotherapy. The brief inter‐modality interval was standardized to maximize potential temporal summation of central and peripheral neuromodulatory effects.

### 2.3 Outcome Measures

The prespecified primary outcome was awakening efficacy at week 4, operationalized a priori using the following three‐tier classification system adjudicated by two blinded physiatrists through daily standardized assessments:

Markedly effective: emergence from coma to a minimally conscious state or higher, accompanied by a ≥3‐point increase in GCS score including ≥1 point on the motor subscale

Effective: demonstration of reproducible command‐following or purposeful behaviors, with a ≥2‐point increase in GCS score
Ineffective: failure to meet the criteria for either of the above categories

Overall awakening efficacy was defined as the proportion of patients achieving either a markedly effective or effective response.

Secondary outcomes encompassed neurophysiological, hemodynamic, and functional domains, and included: (1) change in EEG grade from baseline to week 4 (ordinal scale, Grades I–V; Synek scale); (2) transcranial Doppler (TCD)–derived cerebral blood flow parameters, specifically peak systolic velocity (Vs), end‐diastolic velocity (Vd), and time‐averaged mean velocity (Vm), as well as the pulsatility index (PI), calculated according to the Gosling formula as PI = (Vs − Vd)/Vm; (3) GCS score at week 4 (all randomized patients); and (4) Montreal Cognitive Assessment (MoCA) score at week 4, assessed exclusively in participants who regained sufficient responsiveness to undergo standardized cognitive testing. Safety outcomes, including seizures and other adverse events, were systematically recorded throughout the 4‐week treatment period.

Electroencephalographic assessments were performed using a 19‐channel digital EEG system (Nihon Kohden EEG‐1200, Tokyo, Japan), with electrode placement according to the international 10–20 system. All recordings were conducted under standardized conditions: patients were positioned supine in a quiet environment, with eyes closed, and recordings continued for a minimum of 20 min. Electrode–skin impedances were maintained below 5 kΩ throughout each session. Recordings were subsequently reviewed offline by two independent neurophysiologists, both blinded to treatment allocation, and classified according to the Synek scale, in which Grade I represents normal or near‐normal background activity and Grade V represents electrocerebral silence. Inter‐rater reliability was quantified using weighted Cohen's *κ*, yielding *κ* = 0.83, indicative of strong agreement. EEG assessments were performed at baseline and at the end of week 4.

Cerebral blood flow velocities were measured using transcranial Doppler ultrasonography (DWL Doppler Box X, Compumedics GmbH, Singen, Germany). The M1 segment of the middle cerebral artery (MCA‐M1) was bilaterally insonated through the transtemporal bone window at an insonation depth of 45–65 mm. To minimize physiological variability, all measurements were performed with patients in the supine position following a standardized rest period of at least 30 min. All TCD assessments were conducted by the same trained sonographer, who was blinded to treatment group assignment throughout the study. The following hemodynamic parameters were recorded: time‐averaged mean velocity (Vm), peak systolic velocity (Vs), and end‐diastolic velocity (Vd). The pulsatility index (PI) was subsequently calculated using the Gosling formula: PI = (Vs − Vd)/Vm. To control for known confounders of cerebral blood flow velocity, sedation levels (Richmond Agitation–Sedation Scale), mean arterial pressure (MAP, measured via continuous arterial line or non‐invasive cuff) and end‐tidal CO_2_ (EtCO_2_, obtained by sidestream capnography and used as a non‐invasive surrogate for arterial *p*CO_2_) were documented at the time of each TCD session; data from patients receiving neuromuscular blockade agents at the time of assessment were excluded from that time point's analysis. TCD measurements were obtained at baseline and at the end of week 4.

### 2.4 Data Collection and Statistical Analysis

The primary analysis followed the intention‐to‐treat principle. Awakening efficacy was compared using risk ratios (RRs) with 95% confidence intervals (CIs) and Fisher's exact test. Within‐group shifts in EEG grade were analyzed using the Cochran–Armitage trend test. Between‐group differences in week 4 EEG grade distribution were compared using ordinal logistic regression with treatment group as the predictor. Continuous week 4 outcomes (e.g., GCS, TCD velocities) were compared using ANCOVA with baseline values as covariates; results are reported as adjusted mean differences with 95% CIs. Within‐group changes used paired tests as appropriate. Holm's method controlled multiplicity across secondary endpoints. Prior to performing ANCOVA and paired *t*‐tests, the distributional assumption of normality for all continuous variables (GCS, MoCA, cerebral blood‐flow velocities [Vm, Vs, Vd], pulsatility index, mean arterial pressure, and end‐tidal CO_2_) was formally verified at both baseline and week 4 using the Shapiro–Wilk test, with visual confirmation by quantile–quantile plots. All continuous outcomes satisfied the assumption of approximate normality within each treatment arm (all Shapiro–Wilk *p* > 0.05), supporting the use of parametric inferential methods. Homogeneity of variance was further confirmed using Levene's test (all *p* > 0.05). Two‐sided *p* < 0.05 was considered statistically significant. Analyses were performed in SPSS v26.0 (IBM) and R v4.3.

## 3 Results

### 3.1 Participant Flow and Baseline Characteristics

Eighty‐six patients were randomized to rTMS (*n* = 29), MNS (*n* = 29), or combination therapy (*n* = 28). Baseline demographics, coma duration, injury etiology, and comorbidities were comparable across groups (all *p* > 0.05; Table 1).

**TABLE 1:** Comparison of baseline demographic and clinical characteristics.

Baseline/group	rTMS (*n* = 29)	MNS (*n* = 29)	Combination (*n* = 28)
Age, years	45.11 ± 5.13	44.76 ± 4.46	44.36 ± 4.35
Sex, *n* (%)			
Male	16 (55.17)	14 (48.28)	17 (60.71)
Female	13 (44.83)	15 (51.72)	11 (39.29)
Duration of coma, days	7.52 ± 1.16	7.38 ± 1.27	7.44 ± 1.31
Cause of injury, *n* (%)			
Traffic accident	16 (55.17)	20 (68.97)	17 (60.71)
Physical assault	8 (27.59)	6 (20.69)	9 (32.14)
Other	5 (17.24)	3 (10.34)	2 (7.14)
Comorbidities, *n* (%)			
Diabetes	4 (13.79)	3 (10.34)	4 (14.29)
Hypertension	6 (20.69)	6 (20.69)	6 (21.43)

*Note*: Values are mean ± SD or *n* (%). No significant differences among groups (*p* > 0.05).

### 3.2 Awakening Efficacy

Awakening efficacy at week 4 was 92.9% (26/28) with combination therapy, 65.5% (19/29) with rTMS, and 62.1% (18/29) with MNS (Table 2). Compared with rTMS, combination therapy increased the probability of awakening (risk ratio [RR] 1.42, 95% CI 1.07–1.88; risk difference [RD] +27.3%, 95% CI +7.6% to +47.1%; Fisher's exact *p* = 0.013). Versus MNS, RR 1.50 (95% CI 1.11–2.02; RD +30.8%, 95% CI +10.7% to +50.9%; NNT ≈ 4; Fisher's exact *p* = 0.006). rTMS and MNS did not differ materially (*p* = 1.000).

**TABLE 2:** Awakening efficacy after 4 weeks.

Group	*n*	Markedly effective	Effective	Ineffective	Overall effective
rTMS group	29	11 (37.93)	8 (27.59)	10 (34.48)	19 (65.52)^*^
MNS	29	9 (31.03)	9 (31.03)	11 (37.93)	18 (62.07)^*^
Combination group	28	15 (53.57)	11 (39.29)	2 (7.14)	26 (92.86)

^*^Overall effective = markedly effective + effective.

*Note*: *p* = 0.013 (rTMS vs. Combination group) and *p* = 0.006 (MNS vs. Combination group) by Fisher's exact test; rTMS vs. MNS: *p* = 1.000.

### 3.3 EEG Grading

All groups demonstrated within‐group shifts toward lower‐severity EEG grades from baseline to week 4 (Table 3; within‐group Cochran–Armitage trend tests, all *p* < 0.001). At week 4, the proportion of patients achieving Grade I–II classification was highest in the combination group (71.4%, 20/28), followed by the rTMS group (62.1%, 18/29) and the MNS group (37.9%, 11/29). Ordinal logistic regression with treatment as the predictor demonstrated significant between‐group differences in week 4 EEG grade distribution (overall *p* = 0.008), with the combination group showing significantly more favorable distributions than MNS (*p* = 0.003) and a non‐significant advantage over rTMS (*p* = 0.094). The combination group showed the most favorable overall EEG grade distribution, consistent with its superior awakening efficacy.

**TABLE 3:** EEG grading before and after treatment.

Group	Time	*n*	Grade I	Grade II	Grade III	Grade IV	Grade V
rTMS	Before	29	0	1	12	16	0
rTMS	After	29	4	14	8	3	0
MNS	Before	29	0	2	10	17	0
MNS	After	29	2	9	12	6	0
Combination	Before	28	0	1	13	14	0
Combination	After	28	5	15	6	2	0

*Note*: Counts (*n*). Comparisons performed with *χ* ^2^ test for trend/linear‐by‐linear association. Within‐group trend: all *p* < 0.001. Between‐group week 4 distribution by ordinal logistic regression: overall *p* = 0.008; Combination vs. MNS *p* = 0.003; Combination vs. rTMS *p* = 0.094.

### 3.4 Cerebral Blood‐Flow Parameters

From baseline to week 4, cerebral blood‐flow parameters improved across all groups (Table 4). Vs increased in the rTMS (80.68 → 90.63 cm/s, Δ+9.95), MNS (81.12 → 88.54 cm/s, Δ+7.42), and combination groups (80.47 → 96.39 cm/s, Δ+15.92). Vm similarly increased: rTMS (40.53 → 50.12, Δ+9.59), MNS (40.10 → 49.83, Δ+9.73), and combination (40.22 → 55.49, Δ+15.27). Vd increased in all groups, indicating improved diastolic cerebral perfusion: rTMS (28.35 → 33.46, Δ+5.11), MNS (28.17 → 32.89, Δ+4.72), and combination (28.52 → 37.24, Δ+8.72). The pulsatility index decreased across all groups, consistent with reduced distal cerebrovascular resistance: rTMS (1.29 → 1.14, Δ−0.15), MNS (1.32 → 1.12, Δ−0.20), and combination (1.29 → 1.07, Δ−0.22). At week 4, the combination group demonstrated significantly higher Vm, Vs, and Vd, and significantly lower PI compared with both monotherapy groups (combination vs. rTMS: Vm *p* < 0.001, Vs *p* < 0.001, Vd *p* = 0.002, PI *p* = 0.034; combination vs. MNS: Vm *p* < 0.001, Vs *p* < 0.001, Vd *p* < 0.001, PI *p* = 0.012). To exclude systemic confounding of cerebral blood‐flow velocities, mean arterial pressure (MAP) and end‐tidal CO_2_ (a validated non‐invasive surrogate for arterial *p*CO_2_) were recorded during every TCD session and remained stable and well balanced across the three arms throughout the 4‐week follow‐up evaluations (week 4 MAP: rTMS 89.3 ± 6.1 mmHg, MNS 88.7 ± 5.8 mmHg, combination 89.5 ± 6.4 mmHg, *p* = 0.872; week 4 end‐tidal CO_2_: rTMS 37.4 ± 2.6 mmHg, MNS 37.1 ± 2.8 mmHg, combination 37.6 ± 2.7 mmHg, *p* = 0.785; one‐way ANOVA). These data indicate that the between‐group differences in cerebral hemodynamic indices cannot be attributed to systemic physiological imbalance, supporting a treatment‐related origin of the observed effects.

**TABLE 4:** Cerebral blood‐flow parameters before and after treatment.

Group	Time	*n*	Vm (cm/s)	Vd (cm/s)	Vs (cm/s)	PI
rTMS	Before	29	40.53 ± 3.84	28.35 ± 3.72	80.68 ± 6.86	1.29 ± 0.15
rTMS	After	29	50.12 ± 5.03	33.46 ± 4.18	90.63 ± 5.44	1.14 ± 0.13
MNS	Before	29	40.10 ± 4.12	28.17 ± 3.54	81.12 ± 5.64	1.32 ± 0.16
MNS	After	29	49.83 ± 5.26	32.89 ± 3.96	88.54 ± 5.20	1.12 ± 0.14
Combination	Before	28	40.22 ± 4.26	28.52 ± 3.83	80.47 ± 7.07	1.29 ± 0.17
Combination	After	28	55.49 ± 7.17	37.24 ± 5.06	96.39 ± 6.23	1.07 ± 0.12

*Note*: PI values presented are the group means of individually calculated PI values; values computed from group‐mean velocities may differ slightly due to Jensen's inequality. Within‐group changes from baseline: all paired *t*‐test *p* < 0.001 for Vm, Vs, Vd, and PI. Between‐group ANCOVA at week 4 with baseline as covariate: Combination vs. rTMS – Vm *p* < 0.001, Vs *p* < 0.001, Vd *p* = 0.002, PI *p* = 0.034; Combination vs. MNS – Vm *p* < 0.001, Vs *p* < 0.001, Vd *p* < 0.001, PI *p* = 0.012.

### 3.5 GCS and MoCA Scores

GCS improved within each group (all paired *t*‐test *p* < 0.001; Table 5), with week 4 means of 11.23 ± 1.12 (combination), 9.42 ± 1.15 (rTMS), and 9.66 ± 1.32 (MNS); the corresponding mean changes from baseline were +6.04, +4.19, and +4.50 points, respectively. ANCOVA adjusting for baseline GCS revealed significant between‐group differences at week 4 (overall *p* < 0.001), with pairwise comparisons demonstrating superiority of combination therapy over both rTMS (adjusted mean difference +1.78, 95% CI 1.13–2.42; *p* < 0.001) and MNS (adjusted mean difference +1.54, 95% CI 0.89–2.19; *p* < 0.001), whereas rTMS and MNS did not differ significantly (*p* = 0.428).

**TABLE 5:** GCS scores (all randomized patients).

Group	*n*	Baseline	Week 4
rTMS	29	5.23 ± 0.64	9.42 ± 1.15*
MNS	29	5.16 ± 0.73	9.66 ± 1.32*
Combination	28	5.19 ± 0.62	11.23 ± 1.12*

*Values are mean ± SD. *P* < 0.05 vs. baseline within same group.

*Note*: Paired *t*‐test: all *p* < 0.001. Baseline‐adjusted ANCOVA at week 4: overall *p* < 0.001; Combination vs. rTMS *p* < 0.001; Combination vs. MNS *p* < 0.001; rTMS vs. MNS *p* = 0.428.

MoCA was administered only in participants who became testable (rTMS 19/29 [65.5%], MNS 18/29 [62.1%], combination 26/28 [92.9%]; Table 6). Among those evaluated, week 4 MoCA means were 21.35 ± 2.87 (combination), 18.74 ± 3.26 (rTMS), and 18.22 ± 3.41 (MNS), indicating mild‐to‐moderate cognitive impairment across all groups consistent with early recovery from sTBI‐related coma.

**TABLE 6:** MoCA at Week 4 (testable subset only).

Group	Evaluated	Week 4 MoCA
rTMS	19	18.74 ± 3.26
MNS	18	18.22 ± 3.41
Combination	26	21.35 ± 2.87

*Note*: MoCA was administered only to patients who regained sufficient responsiveness. Values are mean ± SD.

**TABLE 7:** Incidence of adverse events in the three groups.

Group	*n*	Seizures	Intracranial hematoma	Pulmonary infection	Sympathetic hyperexcitability	Total
rTMS	29	0 (0.00)	2 (6.90)	1 (3.45)	3 (10.34)	6 (20.69%)
MNS	29	0 (0.00)	3 (10.34)	2 (6.90)	3 (10.34)	8 (27.59%)
Combination	28	0 (0.00)	1 (3.57)	1 (3.57)	1 (3.57)	3 (10.71%)

*Note*: No statistically significant differences in total adverse event rates were observed between groups (all p > 0.05); differences in adverse event rates were numerically lower in the combination group but did not reach statistical significance, likely owing to insufficient power for safety endpoints. Fisher's exact tests for total adverse event rate: Combination vs. rTMS *p* = 0.471; Combination vs. MNS *p* = 0.180; rTMS vs. MNS *p* = 0.747.

Given the differential testability rates across groups (combination: 92.9%; rTMS: 65.5%; MNS: 62.1%), between‐group comparisons of MoCA scores are subject to substantial selection bias: the testable subset of the combination group constitutes a larger and potentially systematically different population compared with the testable subsets of the monotherapy groups. Accordingly, MoCA findings are designated as exploratory and hypothesis‐generating only and should not be interpreted as confirmatory evidence of superior cognitive benefit with combination therapy.

As a pre‐specified sensitivity analysis to partially mitigate selection bias, MoCA scores were compared exclusively among patients classified as “markedly effective” across all three groups (rTMS: *n* = 11, MNS: *n* = 9, combination: *n* = 15). Within this more homogeneous subgroup, combination therapy continued to show numerically higher MoCA scores (22.13 ± 2.54) compared with rTMS (19.82 ± 2.91) and MNS (19.44 ± 3.05). However, this analysis is underpowered due to small subgroup sizes and should be interpreted with caution.

### 3.6 Adverse Events

No seizures occurred (Table 7). Total AE rates were 10.7% (3/28) for combination, 20.7% (6/29) for rTMS, and 27.6% (8/29) for MNS. The most frequent events were sympathetic hyperexcitability and intracranial hematoma. Relative to rTMS and MNS, AE risk with combination was numerically lower (RR_combo vs. rTMS 0.52, 95% CI 0.14–1.87; Fisher's exact *p* = 0.471; RR_combo vs. MNS 0.39, 95% CI 0.11–1.32; Fisher's exact *p* = 0.180), though CIs were wide due to small counts. All events resolved without sequelae.

## 4 Discussion

This randomized controlled trial demonstrates that combining rTMS with right MNS yields higher awakening efficacy than either modality alone in patients with severe traumatic brain injury and persistent coma. At 4 weeks, 92.9% of patients receiving combination therapy achieved predefined awakening criteria, compared with 65.5% and 62.1% receiving rTMS or MNS alone, respectively. Convergent improvements were observed across EEG grading and TCD‐derived cerebral hemodynamic parameters—including increased blood‐flow velocities and reduced pulsatility index—and functional recovery reflected by GCS also favored the combined approach. In an exploratory analysis limited to patients who became testable, MoCA scores were highest after combined therapy. Importantly, no seizures occurred and adverse events were infrequent across groups.

### 4.1 Potential Mechanisms and Synergy

Recovering consciousness likely requires re‐establishing interactions between cortico‐thalamic and brainstem arousal systems; a dual‐pathway neuromodulation strategy is therefore biologically plausible. rTMS can modulate cortical excitability and reshape large‐scale networks that subserve arousal and attention, supporting synaptic plasticity and network integration (Huang et al. 2023; Jiang et al. 2019). MNS, in contrast, is thought to activate ascending reticular pathways and thalamo‐cortical circuits from the periphery; right‐sided stimulation has been hypothesized to more strongly engage vigilance‐related hemispheric networks. Delivering rTMS and MNS together may thus couple top‐down cortical facilitation with bottom‐up arousal drive, accelerating the network reorganization required for emergence from coma.

Clinical evidence aligns with this mechanistic model. An RCT by Xiong et al. (2023) reported that rTMS+MNS produced greater improvements in consciousness than either treatment alone, measured by CRS‐R and EEG activity. Trials of rTMS monotherapy show variable efficacy, consistent with the notion that cortical stimulation alone may be insufficient in some patients: Fan et al. (2022) found that 20 Hz left DLPFC rTMS improved consciousness scores in a subset compared with sham, while earlier case‐level work by Louise‐Bender Pape et al. (2009) observed neurobehavioral gains during a 6‐week protocol, accompanied by improvements in brainstem auditory evoked potentials that suggest enhanced neural conduction. Reviews similarly emphasize rTMS's capacity to modulate excitability and plasticity but note that clinical applications remain heterogeneous and debated (Huang et al. 2023). Against this backdrop, our randomized comparison extends the literature by isolating the added value of combining rTMS with MNS over either monotherapy, with convergent signals across clinical (awakening efficacy, GCS, exploratory MoCA) and neurophysiological/hemodynamic readouts (EEG grading and TCD‐derived cerebral blood‐flow velocities and pulsatility index), thereby reinforcing a synergy between central and peripheral neuromodulation.

### 4.2 Comparison With Prior Studies

#### 4.2.1 Comparison With Prior Studies on rTMS Monotherapy

The 65.5% awakening efficacy observed in our rTMS group is broadly consistent with, though at the higher end of, outcomes reported in prior controlled investigations. The meta‐analysis by Yang et al. (2023), encompassing 207 patients from seven randomized trials, demonstrated that rTMS significantly improved CRS‐R scores in DoC patients versus controls (WMD = 1.89, 95% CI: 1.39–2.39; *p* < 0.00001), with subgroup analyses confirming significant effects for DLPFC stimulation (WMD = 2.24; *I* ^2^ = 31%) and protocols exceeding 20 sessions (WMD = 1.75; *I* ^2^ = 12%). Although that meta‐analysis reported continuous CRS‐R changes rather than categorical awakening rates, the magnitude of improvement is directionally compatible with our findings. Our modestly higher categorical awakening rate may reflect the homogeneous sTBI population (GCS ≤ 8), earlier enrollment (mean coma duration ∼7.4 days, when neural plasticity is presumably highest), and the use of a composite awakening criterion that may capture a broader spectrum of recovery than CRS‐R total score alone. Fan et al. (2022), in a randomized double‐blinded sham‐controlled trial of 40 DoC patients, found that 20 Hz left DLPFC rTMS at 100% RMT significantly improved CRS‐R scores versus sham (8.45 ± 3.55 vs. 6.25 ± 1.29), but only in a subset of patients, and rTMS did not significantly enhance the overall awakening ratio. The discrepancy with our higher categorical awakening rate likely reflects key methodological differences: their cohort included mixed etiologies with substantially longer time since onset (∼49 days vs. ∼7 days), different stimulation parameters (20 Hz at 100% RMT vs. our 10 Hz at 80% RMT), and different outcome operationalizations. Louise‐Bender Pape et al. (2009) provided early safety and proof‐of‐concept data in a single vegetative‐state patient (287 days post‐TBI), demonstrating a trend toward neurobehavioral gains (*p* = 0.066) with 6‐week right DLPFC rTMS and concurrent improvements in brainstem auditory evoked potentials; however, the single‐subject design and different stimulation target preclude direct efficacy comparison. Collectively, these studies support the therapeutic potential of rTMS in DoC while highlighting the substantial influence of patient selection, time since injury, stimulation parameters, and outcome measurement on reported efficacy.

#### 4.2.2 Comparison With Prior Studies on MNS Monotherapy

The 62.1% awakening rate in our MNS group should be contextualized within an evidence base that remains limited and inconclusive. The systematic review by Feller et al. (2021) identified five randomized controlled trials of MNS in DoC but concluded that no definitive determination of effectiveness could be made, owing to the small number of eligible studies and overall high risk of bias. Nevertheless, preliminary signals of benefit were noted: one included trial reported a higher percentage of patients regaining consciousness at six months in the MNS group, and another demonstrated reduced ICU hospitalization days. Our finding of 62.1% awakening under a standardized protocol (right MNS, 20 Hz, 300 µs, 20 min/session, 20 sessions over 4 weeks) contributes controlled data to this evidence‐sparse area and is broadly consistent with the direction—though not the certainty—of benefit suggested by these earlier trials. Variability in MNS outcomes across the literature likely reflects differences in stimulation laterality, intensity calibration, treatment duration, and the heterogeneity of underlying lesion patterns affecting ascending somatosensory and reticular arousal pathway integrity.

#### 4.2.3 Comparison With Combination Therapy Evidence

The 92.9% awakening efficacy in our combination group is broadly consistent with Xiong et al. (2023), who randomized 75 prolonged DoC patients (traumatic and non‐traumatic) to MNS + rTMS, rTMS alone, or MNS alone. Their combination group showed significantly greater CRS‐R, GCS, EEG, and N20 improvements versus either monotherapy, though notably the awakening ratios did not differ significantly between groups. The discrepancy with our higher categorical awakening rate likely reflects key differences: our cohort comprised exclusively acute‐phase sTBI patients (mean coma duration ∼7.4 days vs. prolonged DoC), with presumably greater residual neural plasticity; stimulation parameters differed (90% vs. 80% RMT; fixed current vs. functionally titrated MNS); and our composite awakening criterion may be more sensitive to partial recovery than their categorical awakening ratio. Nevertheless, both studies converge on the conclusion that combined MNS + rTMS yields superior outcomes over either monotherapy, reinforcing the dual‐pathway neuromodulation rationale.

#### 4.2.4 Comparison With Pharmacologic Approaches

Relative to pharmacologic interventions, Giacino et al. (2012) randomized 184 post‐traumatic DoC patients (VS and MCS, 4–16 weeks post‐injury) to amantadine or placebo for 4 weeks. Amantadine significantly accelerated DRS‐measured functional recovery during active treatment (difference in slope, 0.24 points/week; *p* = 0.007), but this advantage reversed during the 2‐week washout period, with overall improvement at week 6 similar between groups. Our combination neuromodulation approach, which targets both cortical and subcortical arousal pathways, may offer complementary or potentially more durable benefits; however, direct comparison is limited by differences in patient populations (acute sTBI with GCS ≤ 8 vs. VS/MCS at 4–16 weeks), outcome instruments (DRS vs. composite awakening criterion), and the absence of post‐treatment follow‐up in our study. Future trials incorporating extended follow‐up are needed to determine whether the benefits of combination neuromodulation persist beyond the active treatment period.

#### 4.2.5 Sources of Heterogeneity Across the Neuromodulation Literature

The substantial heterogeneity observed across neuromodulation studies in DoC likely reflects the interplay of several factors: (1) variability in stimulation parameters, including frequency, intensity, total pulse count, and cortical target localization method; (2) differences in patient populations with respect to injury etiology, time since injury, baseline consciousness level, and lesion location and extent; (3) inconsistent outcome operationalization, ranging from continuous CRS‐R score changes to categorical awakening criteria to GCS‐based endpoints; and (4) common methodological limitations such as small sample sizes, absence of sham controls, and lack of assessor blinding. Our study addresses several of these limitations through its three‐arm randomized design, standardized and reproducible protocols, assessor blinding, and multimodal outcome assessment spanning clinical, neurophysiological, and hemodynamic domains. Nevertheless, we share the common constraints of modest sample size, single‐center design, and limited follow‐up duration. Establishing international consensus on minimum reporting standards, core outcome sets, and stimulation parameter reporting for neuromodulation trials in DoC would greatly facilitate evidence synthesis and accelerate clinical translation. Future multicenter trials with larger sample sizes, standardized outcome measures (CRS‐R, GOSE), extended follow‐up (3–6 months), and dose‐finding designs are needed to confirm our findings, determine the durability of treatment effects, and identify optimal stimulation parameters and candidate patient profiles for combination neuromodulation therapy.

### 4.3 Clinical Implications

The absolute difference of ∼27–31 percentage points in awakening efficacy suggests that combined noninvasive neuromodulation could provide clinically meaningful benefits when added to standard rehabilitation. Protocols appear feasible (20‑min sessions, 5 days/week) and were well tolerated in this cohort. Implementation would require standardized stimulation parameters, assessor blinding, and safety monitoring (particularly for dysautonomia and intracranial complications), but the absence of seizures is reassuring.

### 4.4 Strengths and Limitations

Strengths include randomization, multimodal outcomes (EEG, TCD, functional), and systematic safety capture. Although a post hoc power estimate is reported in Section 2 for transparency, we acknowledge that such calculations have recognized statistical limitations and should not substitute for the a priori sample size justification provided therein. This study has several important limitations. First, the single‐center design and modest sample size (*n* = 86) limit generalizability; replication in multicenter settings with diverse patient populations is essential. Second, the 4‐week observation window precludes assessment of the durability of treatment effects; future studies should include extended follow‐up at 3, 6, and 12 months using standardized instruments such as the CRS‐R and GOSE. Third, and critically, the absence of a sham‐stimulation or standard‐care‐only control arm means that the observed improvements in all three groups may partly reflect natural neurological recovery during the acute‐to‐subacute phase, which can be substantial in sTBI patients with relatively short coma durations. The between‐group differences likely reflect differential treatment effects, but the absolute magnitude of improvement attributable to neuromodulation per se cannot be isolated without a no‐active‐treatment comparator. Fourth, although outcome assessors were blinded, patients and treating clinicians were necessarily unblinded due to the perceptible nature of both rTMS and MNS, and formal blinding integrity assessment was not performed. Fifth, the use of MoCA only in a testable subset—whose composition was determined by the primary outcome itself—introduces informative selection bias that precludes valid between‐group cognitive comparisons; the complete overlap between awakened and testable patients further underscores this limitation. Future trials should employ the CRS‐R or other consciousness‐level instruments as primary outcomes applicable to all patients, with cognitive assessments reserved for pre‐specified secondary analyses at later recovery time points. Statistical approaches to address informative missingness (e.g., multiple imputation under missing‐not‐at‐random assumptions, inverse probability weighting, worst‐rank composites) should be incorporated into the analytical framework. Sixth, although post hoc power was estimated at approximately 87% for the primary outcome, post hoc power calculations are inherently limited in interpretive value; the a priori calculation remains the primary basis for judging study adequacy. Seventh, TCD‐derived blood flow velocity is an indirect measure of cerebral perfusion; complementary modalities such as CT perfusion or arterial spin labeling MRI would strengthen hemodynamic conclusions. Finally, the trial was not powered for safety endpoints, and the numerically lower adverse event rate in the combination group should not be interpreted as evidence of superior safety.

## 5 Conclusions

In patients with sTBI and persistent coma, rTMS combined with right MNS improved awakening efficacy and neurophysiologic/functional indices over either modality alone, with low rates of adverse events. Confirmation in multicenter trials with longer follow‑up, assessor blinding, standardized TCD protocols, and effect size reporting is warranted. Dose‑finding and personalization based on EEG/TCD biomarkers may further optimize treatment.

## Author Contributions

**Manrong Li**: methodology, investigation, formal analysis, Writing – review and editing. **Nana Wang**: investigation, data curation, formal analysis, visualization, writing – original draft. **Guozhen Zhang**: conceptualization, methodology, resources, supervision, project administration.

## Funding

The authors have nothing to report.

## Ethics Statement

This study was approved by the Institutional Review Board of Shanghai Xinqidian Rehabilitation Hospital. Because participants were comatose, written informed consent was obtained from their legal representatives prior to enrollment. All procedures adhered to the Declaration of Helsinki and relevant national regulations.

## Consent for Publication

Consent for publication of de‐identified clinical data was obtained from the legal representatives of all participants.

## Conflicts of Interest

The authors declare no competing interests.

## AI Usage Declaration

The authors declare that no generative artificial intelligence (AI) tools or AI‐assisted technologies (including, but not limited to, large language models such as ChatGPT, or automated text‐generation, image‐generation, or data‐analysis software) were used in the conception, design, data collection, data analysis, interpretation of results, or the writing, drafting, editing, or revision of this manuscript. All content presented in this manuscript is the original intellectual work of the listed authors, who take full responsibility for its accuracy and integrity.

## Data Availability

The results reported in this article will be made available upon reasonable request to the corresponding author.

## References

[brb371661-bib-0001] Cossu, G. 2014. “Therapeutic Options to Enhance Coma Arousal After Traumatic Brain Injury: state of the Art of Current Treatments to Improve Coma Recovery.” British Journal of Neurosurgery 28, no. 2: 187–198. 10.3109/02688697.2013.841845. 24090192

[brb371661-bib-0002] Dewan, M. C. , A. Rattani , S. Gupta , et al. 2019. “Estimating the Global Incidence of Traumatic Brain Injury.” Journal of Neurosurgery 130, no. 4: 1080–1097. 10.3171/2017.10.JNS17352. 29701556

[brb371661-bib-0003] Du, Q. , L. Huang , Y. Tang , J. Kang , W. Ye , and Z. Feng . 2022. “Median Nerve Stimulation Attenuates Traumatic Brain Injury–Induced Comatose State by Regulating the Orexin‐A/RasGRF1 Signaling Pathway.” World Neurosurgery 168: e19–e27. 10.1016/j.wneu.2022.07.085. 36064116

[brb371661-bib-0004] Fan, J. , Y. Zhong , H. Wang , N. Aierken , and R. He . 2022. “Repetitive Transcranial Magnetic Stimulation Improves Consciousness in Some Patients With Disorders of Consciousness.” Clinical Rehabilitation 36, no. 7: 916–925. 10.1177/02692155221089455. 35322709

[brb371661-bib-0005] Feller, D. , C. Vinante , F. Trentin , and T. Innocenti . 2021. “The Effectiveness of Median Nerve Electrical Stimulation in Patients With Disorders of Consciousness: A Systematic Review.” Brain Injury 35, no. 4: 385–394. 10.1080/02699052.2021.1887522. 33617359

[brb371661-bib-0006] Giacino, J. T. , J. Whyte , E. Bagiella , et al. 2012. “Placebo‐Controlled Trial of amantadine for Severe Traumatic Brain Injury.” New England Journal of Medicine 366, no. 9: 819–826. 10.1056/NEJMoa1102609. 22375973

[brb371661-bib-0007] Hallett, M. 2007. “Transcranial Magnetic Stimulation: A Primer.” Neuron 55, no. 2: 187–199. 10.1016/j.neuron.2007.06.026. 17640522

[brb371661-bib-0008] Huang, W. , Q. Chen , J. Liu et al. 2023. “Transcranial Magnetic Stimulation in Disorders of Consciousness: An Update and Perspectives.” Aging and disease 14, no. 4: 1171–1183. 10.14336/AD.2022.1114 PMC10389824 37163434

[brb371661-bib-0009] Huang, Y.‐Z.u , M. J. Edwards , E. Rounis , K. P. Bhatia , and J. C. Rothwell . 2005. “Theta Burst Stimulation of the Human Motor Cortex.” Neuron 45, no. 2: 201–206. 10.1016/j.neuron.2004.12.033. 15664172

[brb371661-bib-0010] Iaccarino, C. , A. Carretta , F. Nicolosi , and C. Morselli . 2018. “Epidemiology of Severe Traumatic Brain Injury.” Journal of Neurosurgical Sciences 62, no. 5: 535–541. 10.23736/S0390-5616.18.04532-0. 30182649

[brb371661-bib-0011] Jiang, Y.i , Z. Guo , G. Xing , et al. 2019. “Effects of High‐Frequency Transcranial Magnetic Stimulation for Cognitive Deficit in Schizophrenia: A Meta‐Analysis.” Frontiers in Psychiatry 10: 135. 10.3389/fpsyt.2019.00135. PMC6450172 30984036

[brb371661-bib-0012] Li, S. , A. L. Zaninotto , I. S. Neville , W. S. Paiva , D. Nunn , and F. Fregni . 2015. “Clinical Utility of Brain Stimulation Modalities Following Traumatic Brain Injury: Current Evidence.” Neuropsychiatric disease and treatment 11: 1573–1586. 10.2147/NDT.S65816 PMC4494620 26170670

[brb371661-bib-0013] Li, Y. , X. Luo , M. Wan , et al. 2018. “The Effectiveness of Non‐invasive Brain Stimulation on Arousal and Alertness in Patients in Coma or Persistent Vegetative state After Traumatic Brain Injury: Protocol of Systematic Review and Network Meta‐Analysis.” Medicine 97, no. 37: e12321. 10.1097/MD.0000000000012321. PMC6155968 30212977

[brb371661-bib-0014] Louise‐Bender Pape, T. , J. Rosenow , G. Lewis , et al. 2009. “Repetitive Transcranial Magnetic Stimulation‐Associated Neurobehavioral Gains During Coma Recovery.” Brain Stimulation 2, no. 1: 22–35. 10.1016/j.brs.2008.09.004. 20633400

[brb371661-bib-0015] Nardone, R. , L. Sebastianelli , V. Versace , et al. 2020. “Repetitive Transcranial Magnetic Stimulation in Traumatic Brain Injury: Evidence From Animal and Human Studies.” Brain Research Bulletin 159: 44–52. 10.1016/j.brainresbull.2020.03.016. 32251693

[brb371661-bib-0016] Neville, I. S. , A. L. Zaninotto , C. Y. Hayashi , et al. 2019. “Repetitive TMS Does Not Improve Cognition in Patients With TBI.” Neurology 93, no. 2: e190–e199. 10.1212/WNL.0000000000007748. PMC6656650 31175209

[brb371661-bib-0017] Schnakers, C. , and M. M. Monti . 2017. “Disorders of Consciousness After Severe Brain Injury: Therapeutic Options.” Current Opinion in Neurology 30, no. 6: 573–579. 10.1097/WCO.0000000000000495. 28901969

[brb371661-bib-0018] Thibaut, A. , M.‐A. Bruno , D. Ledoux , A. Demertzi , and S. Laureys . 2014. “tDCS in Patients With Disorders of Consciousness: Sham‐Controlled Randomized Double‐Blind Study.” Neurology 82, no. 13: 1112–1118. 10.1212/WNL.0000000000000260. 24574549

[brb371661-bib-0019] Wang, Y. , C. Huang , R. Tian , and X.i Yang . 2023. “Target Temperature Management and Therapeutic Hypothermia in Sever Neuroprotection for Traumatic Brain Injury: Clinic Value and Effect on Oxidative Stress.” Medicine 102, no. 10: e32921. 10.1097/MD.0000000000032921. PMC9997789 36897685

[brb371661-bib-0020] Whyte, J. , and R. Myers . 2009. “Incidence of Clinically Significant Responses to zolpidem Among Patients With Disorders of Consciousness: A Preliminary Placebo Controlled Trial.” American Journal of Physical Medicine & Rehabilitation 88, no. 5: 410–418. 10.1097/PHM.0b013e3181a0e3a0. 19620954

[brb371661-bib-0021] Xiong, Q.i , K. Le , Y. Tang , et al. 2023. “Effect of Single and Combined Median Nerve Stimulation and Repetitive Transcranial Magnetic Stimulation in Patients With Prolonged Disorders of Consciousness: A Prospective, Randomized, Single‐Blinded, Controlled Trial.” Frontiers in Aging Neuroscience 15: 1112768. 10.3389/fnagi.2023.1112768. PMC10164991 37168716

[brb371661-bib-0022] Yan, X.‐J. , C.‐P. Zhan , Y. Lv , et al. 2022. “Utility of Serum Nuclear Factor Erythroid 2‐Related Factor 2 as a Potential Prognostic Biomarker of Severe Traumatic Brain Injury in Adults: A Prospective Cohort Study.” Frontiers in Neurology 13: 1013062. 10.3389/fneur.2022.1013062. PMC9663921 36388174

[brb371661-bib-0023] Yang, Z. , T. Yue , V. R. Zschorlich , D. Li , D. Wang , and F. Qi . 2023. “Behavioral Effects of Repetitive Transcranial Magnetic Stimulation in Disorders of Consciousness: A Systematic Review and Meta‐Analysis.” Brain Sciences 13, no. 10: 1362. 10.3390/brainsci13101362. PMC10605911 37891731

[brb371661-bib-0024] Zhao, H.e‐X. , Y.i Liao , D. Xu , et al. 2016. “Prospective Randomized Evaluation of Therapeutic Decompressive Craniectomy in Severe Traumatic Brain Injury With Mass Lesions (PRECIS): Study Protocol for a Controlled Trial.” BMC Neurology 16: 1. 10.1186/s12883-015-0524-9. PMC4700654 26727957

[brb371661-bib-0025] Zhong, Y.‐J. , Z. Feng , L. Wang , and T.‐Q.i Wei . 2015. “Wake‐Promoting Actions of Median Nerve Stimulation in TBI‐Induced Coma: An Investigation of Orexin‐A and Orexin Receptor 1 in the Hypothalamic Region.” Molecular Medicine Reports 12, no. 3: 4441–4447. 10.3892/mmr.2015.3898. 26059340

